# Immunohistochemical Study of the PD-1/PD-L1 Pathway in Cutaneous Lupus Erythematosus

**DOI:** 10.3389/pore.2022.1610521

**Published:** 2022-08-01

**Authors:** Zsófia Király, Ágota Szepesi, Anna Sebestyén, Enikő Kuroli, Fanni Rencz, Béla Tóth, Laura Bokor, József Szakonyi, Márta Medvecz, Bernadett Hidvégi

**Affiliations:** ^1^ Department of Dermatology, Venereology and Dermatooncology, Semmelweis University, Budapest, Hungary; ^2^ 1st Department of Pathology and Experimental Cancer Research, Semmelweis University, Budapest, Hungary; ^3^ Department of Health Economics, Corvinus University of Budapest, Budapest, Hungary

**Keywords:** PD-L1, PD-1, subacute cutaneous lupus erythematosus, discoid lupus erythematosus, PD-1 inhibitor-induced SCLE, TEN-like lupus

## Abstract

The pathomechanism of various autoimmune diseases is known to be associated with the altered function of programmed cell death 1/programmed cell death ligand 1 (PD-1/PD-L1) axis. We aimed to investigate the role of this pathway and inflammatory cell markers in subtypes of cutaneous lupus erythematosus (CLE): discoid lupus erythematosus (DLE), subacute CLE (SCLE) and toxic epidermal necrolysis (TEN)-like lupus, a hyperacute form of acute CLE (ACLE). Ten skin biopsy samples from 9 patients were analyzed with immunohistochemistry regarding the following markers: CD3, CD4, CD8, Granzyme B, CD123, CD163, PD-1, PD-L1. Our group consisted of 4 SCLE (2 idiopathic (I-SCLE) and 2 PD-1 inhibitor-induced (DI-SCLE)), 4 DLE and 1 TEN-like lupus cases. From the latter patient two consecutive biopsies were obtained 1 week apart. Marker expression patterns were compared through descriptive analysis. Higher median keratinocyte (KC) PD-L1 expression was observed in the SCLE group compared to the DLE group (65% and 5%, respectively). Medians of dermal CD4, Granzyme B (GB), PD-1 positive cell numbers and GB+/CD8^+^ ratio were higher in the DLE group than in the SCLE group. The I-SCLE and DI-SCLE cases showed many similarities, however KC PD-L1 expression and dermal GB positive cell number was higher in the former. The consecutive samples of the TEN-like lupus patient showed an increase by time within the number of infiltrating GB+ cytotoxic T-cells and KC PD-L1 expression (from 22 to 43 and 30%–70%, respectively). Alterations of the PD-1/PD-L1 axis seems to play a role in the pathogenesis of CLE.

## Introduction

The programmed cell death 1/programmed cell death ligand 1 (PD-1/PD-L1) pathway is one of the numerous cell signaling mechanisms that regulate complex immunological communication between immune and parenchymal cells. The PD-1/PD-L1 axis is known to affect the pathomechanism of autoimmunity and the outcome of various types of cancers through regulation of innate and adaptive immune responses (1).

Immune checkpoint inhibitors are commonly used to treat patients with cancer. Targets of these therapies are the PD-1 receptor and its ligand, PD-L1, two key molecules in T-cell signaling pathways (2). PD-1/PD-L1 inhibitors enhance immune reactivity by activating T-cells and blocking their apoptosis. Due to the enhanced T-cell response various autoimmune adverse side effects might occur during these therapies, many of which are skin-related: vitiligo and lichenoid dermatitis are common ones, while bullous pemphigoid, psoriasis or subacute cutaneous lupus erythematosus (SCLE) occur less frequent (3, 4). Case reports have recently been published on PD-1 inhibitor (nivolumab and pembrolizumab) induced SCLE, but the definite mechanism behind this process is still unclear (5–7). To date, no study has investigated the role of PD-1/PD-L1 pathway in any subtype of cutaneous lupus erythematosus (CLE).

Clinical characteristics of SCLE are erythematous, scaling, papulosquamous or annular eruptions, usually presenting in sun-exposed areas, with no tendency for scarring. Up to 50% of SCLE cases fulfil the diagnostic criteria of systemic lupus erythematosus (SLE) (8, 9). Some drugs (e.g., antihypertensives, terbinafine, proton pump inhibitors, immune checkpoint inhibitors) can induce SCLE, which presents with a hardly different clinical picture as the idiopathic form. Since the pathogenesis of SCLE is probably multifactorial and involves several hits, differentiating the drug-induced and the idiopathic form can be uneasy (10, 11). Beside SCLE acute CLE (ACLE) and chronic CLE (CCLE) are other subtypes of CLE (12). ACLE might present as malar rash or erythematous macules and papules widespread and has a very strong association with SLE (12). Discoid lupus erythematosus (DLE) is a common type of CCLE, it is characterized by livid erythematous, deeply infiltrated, scarring plaques, absence of any autobody and rare association with SLE (8, 13).

This study aims to investigate the possible histopathological similarities and alterations between DLE and SCLE regarding the PD-1/PD-L1 pathway. Another objective was to explore the possible differences between PD-1 inhibitor provoked and idiopathic SCLE. We expanded our study to investigate a rare case of a hyperacute form of ACLE called toxic epidermal necrolysis (TEN)-like lupus. This case allowed us to analyze changes of the PD-1/PD-L1 axis in timeline.

## Materials and Methods

### Patients and Skin Biopsy Samples

Ten skin biopsy samples were collected from 9 patients who presented at the Department of Dermatology, Venereology and Dermatooncology of Semmelweis University. This study involving human participants was in accordance with the 1964 Helsinki Declaration and its later amendments or comparable ethical standards. Ethical approval was waived by the Regional Institutional Scientific and Research Committee of Semmelweis University, Budapest, Hungary (licence number: 5/2021, date of approval: 24 February 2021) in view of the retrospective nature of the study and all the procedures being performed were part of the routine care.

Four patients were diagnosed with SCLE, 2 of them were induced with PD-1 inhibitor therapy administered for malignant melanoma (DI-SCLE) (nivolumab 6th dose, pembrolizumab 1st dose), 2 of them had no provoking drug in their history (I-SCLE). Four of our patients had DLE, while 1 patient had a history of SLE and developed TEN-like lupus (no history of provoking drug). From the latter patient, 2 biopsies were taken at different time points due to rapid progression of skin alterations: one at beginning of the skin symptoms and the second time 1 week later at the time of skin detachment.

### Immunohistochemistry

The LEICA Bond Max fully automatized staining system was used for immunohistochemistry. For antigen localization Bond Polymer Refine Detection kit was used (LEICA). The antibodies employed recognized the following antigens: PD-L1 (DAKO, clone 22C3), PD-1 (Cell Marque, clone NAT105), CD3 (DAKO, clone A0452), CD4 (LEICA, clone NCL-L-CD4-368), CD8 (BioSB, clone EP334), Granzyme B (GB) (DAKO, clone Ret40f), CD123 (Cell Marque, clone 6H6), CD163 (LEICA, clone 10D6).

The procedures were done according to the instructions provided by the manufacturer. Special antigen retrieval was applied for PD-L1 staining in pressure cooker for 25 min with DAKO retrieval solution (Target Retrieval Solution Low pH), incubation time with the primary antigen was 2 × 60 min. Tonsil and normal skin samples were utilized as positive and negative controls.

Stained slides were scanned at ×40 magnification using a Pannoramic scan instrument (3D Histech, Budapest, Hungary) equipped with a Carl Zeiss objective (NA = 0.83; Carl Zeiss MicroImaging Inc., Jena, Germany). The relative proportion of the positive keratinocytes (KCs) and numbers of inflammatory cells were determined on the scanned slides. KC PD-L1, CD123 and CD163 stainings were evaluated in the whole sample. PD-L1 staining of the KCs was considered positive if there was moderate to high intensity staining in >1% of the cells. CD123 positive plasmacytoid dendritic cells (pDCs) were counted in the dermis and were evaluated as 1–5% 1+, 6–15% 2+ and 16–30% 3+. CD163 positive histiocytes were counted in the dermis and were evaluated as 1–20% 1+, 21–50% 2+ and >50% 3+. The number of PD-1, PD-L1, CD3, CD4, CD8, and GB positive lymphocytes were counted in the most diagnostic, 7 × 3–4 mm wide area of the sample in ×20 magnification in the dermis by two independent pathologists.

### Data Analysis

The different types of CLE were compared through descriptive analysis of the immunohistochemical results. Case no. 9 and 10 were excluded from this part due to the multiorgan involvement of the patient. Statistical tests were not carried out due to the small sample size.

## Results

### Clinical and Histopathological Data

Six female and three male patients between ages 37 and 78 years at the time of diagnosis were included in the study. The clinicopathological characteristics of the patients are summarized in Table 1.

**TABLE 1:** Clinicopathological characteristics of our patients.

Case no	Sex/age at diagnosis	Diagnosis	Localisation	Antibodies
1	M/78	DI-SCLE (nivolumab 6th dose)	Chest, arms, back	ANA, SSA
2	F/71	DI-SCLE (pembrolizumab 1st dose)	Arms	ANA
3	F/72	I-SCLE	Neck, V-line, arms, back	ANA, SSA
4	F/77	I-SCLE	Face, chest, arms, shoulders, back, buttocks, legs	ANA, SSA, SSB, RF
5	M/43	DLE	Nose	None
6	M/37	DLE	Face	None
7	F/31	DLE	Face, back	None
8	F/45	DLE	Face, back, scalp	None
9	F/72	TEN-like lupus	Widespread	ANA, dsDNA
10				

M, male; F, female; SCLE, subacute cutaneous lupus erythematosus; DI-SCLE, PD-1 inhibitor-induced SCLE; I-SCLE, idiopathic SCLE; DLE, discoid lupus erythematosus; TEN, toxic epidermal necrolysis; ANA, anti-nuclear antibody; SSA, anti-Sjögren’s-syndrome-related antigen A; SSB, anti-Sjögren’s-syndrome-related antigen B; RF, rheuma factor; dsDNA, double stranded deoxyribonucleic acid.

**FIGURE 1:**
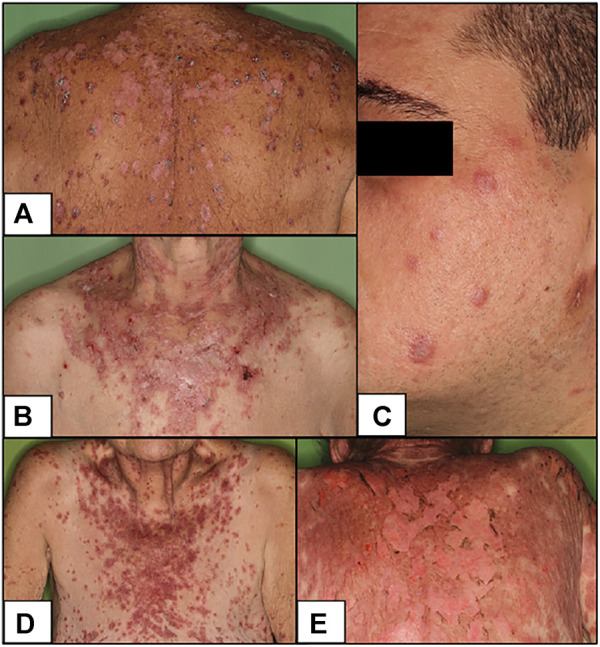
Clinical pictures of our patients **(A)** DI-SCLE (Case 1) **(B)** I-SCLE (Case 3) **(C)** DLE (Case 6) **(D,E)** TEN-like (ACLE) lupus early and late (Case 9 and 10) Clinical presentations of DI-SCLE and SCLE cases reveal similarities: scaling, erythematous plaques in sun-exposed body parts. Clinical picture of DLE shows the deeply infiltrated, discoid plaques on the face. The two pictures of the TEN-like lupus case present the progression of the clinical picture (SCLE, subacute cutaneous lupus erythematosus; DI-SCLE, PD-1 inhibitor-induced SCLE; I-SCLE, idiopathic SCLE; DLE, discoid lupus erythematosus; TEN, toxic epidermal necrolysis).

The localizations of their symptoms differed: for SCLE patients sun-exposed areas, while for DLE patients facial involvement were characteristic (Figure 1). SCLE and TEN-like lupus cases tested positive for anti-nuclear antibody (ANA), both idiopathic SCLE patients had anti-Sjögren’s-syndrome-related antigen A (SSA) and one of them also had anti-Sjögren’s-syndrome-related antigen B (SSB) and rheuma factor (RF), and one DI-SCLE patient had SSA. The TEN-like lupus patient showed positivity for anti double stranded deoxyribonucleic acid (anti-dsDNA). Antibody profile of DLE cases showed no presence of any antibody.

Histological analysis of the samples revealed different stages of interface dermatitis in all cases. In DLE a more prominent follicular plugging and perifollicular lymphocytic infiltrate were found compared to SCLE cases, where this localization was uncommon.

### Immunohistochemistry

**FIGURE 2:**
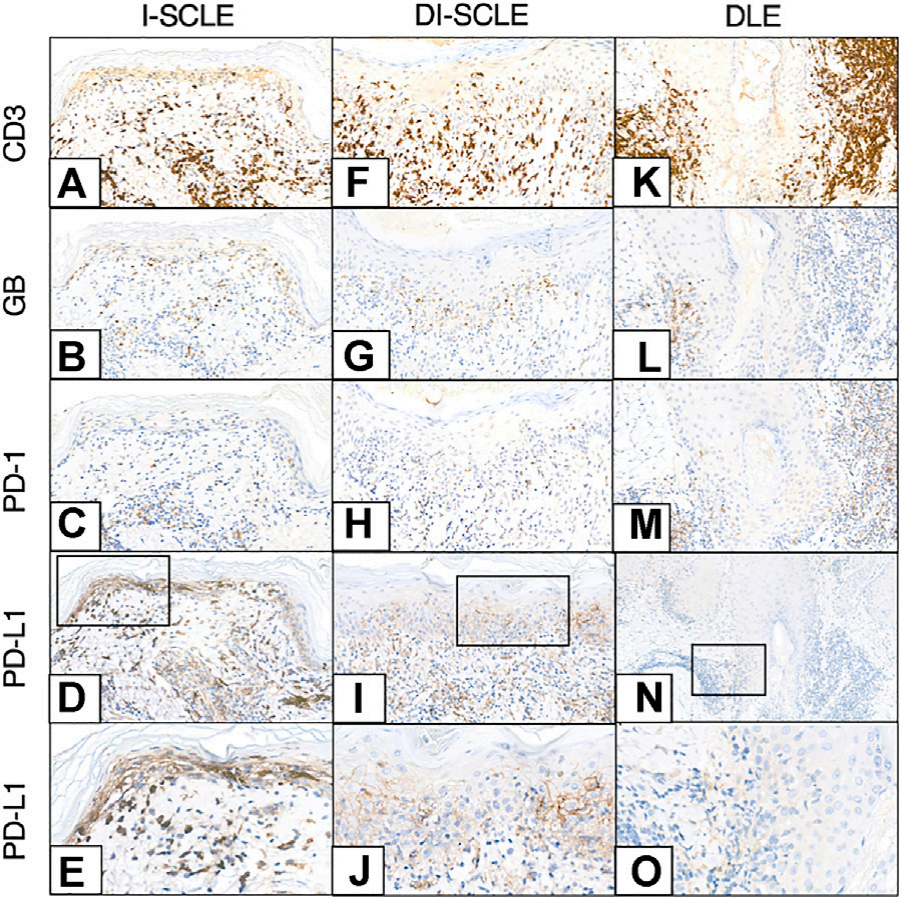
CD3, GB, PD-1, PD-L1 immunohistochemistry of I-SCLE, DI-SCLE and DLE **(A–E) I-**SCLE **(F–J)** DI-SCLE **(K–O)** DLE [Magnifications: ×20, except: **(N)**, ×10; **(E,J,O)**: ×40] The immunohistochemical pictures of DI-SCLE and I-SCLE do not show many differences, however dermal GB cell number is higher in I-SCLE. In DLE a more massive and perifollicular dermal infiltrate can be observed compared to SCLEs. KC PD-L1 expression is elevated in all cases, however a high increase can be seen in SCLEs (regardless of origin) while only slight increase in DLE (SCLE, subacute cutaneous lupus erythematosus; DI-SCLE, PD-1 inhibitor-induced SCLE; I-SCLE, idiopathic SCLE; DLE, discoid lupus erythematosus; GB, granzyme B; KC, keratinocyte; PD-1, programmed cell death 1; PD-L1, programmed cell death ligand 1).

CD3, GB, PD-1 and PD-L1 immunostainings of SCLE and DLE cases are shown in Figure 2.

PD-L1 expression of the epidermal KCs was present in all 10 samples compared to normal skin, where no positive staining was observed. The results of PD-L1 immunostainings are summarized in Table 2.

**TABLE 2:** Results of PD-L1 immunohistochemistry.

Case no	Diagnosis	Epidermis	Dermis									
		KC PD-L1 (%)	CD3 (count/field)	CD4 (count/field)	CD8 (count/field)	GB (count/field)	GB/CD8 ratio	PD-1 (count/field)	PD-1/CD3 ratio	PD-L1 (count/field)	CD123	CD163
1	DI-SCLE	40	360	160	120	25	0.21	12	0.03	65	2+	2+
2	DI-SCLE	50	80	18	66	13	0.20	8	0.10	68	1+	3+
3	I-SCLE	80	280	102	230	54	0.23	48	0.17	105	2+	1+
4	I-SCLE	80	145	20	78	41	0.53	12	0.08	28	3+	2+
SCLE group median		65	212.5	61	99	33	0.22	12	0.09	66.5		
5	DLE	5	260	190	78	48	0.62	55	0.21	38	1+	2+
6	DLE	5	400	235	140	68	0.49	21	0.05	90	3+	2+
7	DLE	5	110	10	90	35	0.39	20	0.18	22	2+	1+
8	DLE	30	230	40	180	79	0.44	40	0.17	90	3+	2+
DLE group median		5	245	115	115	58	0.46	30.5	0.18	64		
9	TEN-like lupus (early)	30	138	18	87	22	0.24	45	0.12	68	—	3+
10	TEN-like lupus (late)	70	108	26	90	43	0.49	27	0.20	13	—	3+

KC PD-L1 stainings were evaluated in the whole sample and were considered positive if there was moderate to high intensity staining in >1% of the cells (SCLE, subacute cutaneous lupus erythematosus; DI-SCLE, PD-1 inhibitor-induced SCLE; DLE, discoid lupus erythematosus; TEN, toxic epidermal necrolysis; KC, keratinocyte; PD-L1, programmed cell death ligand 1).

**FIGURE 3:**
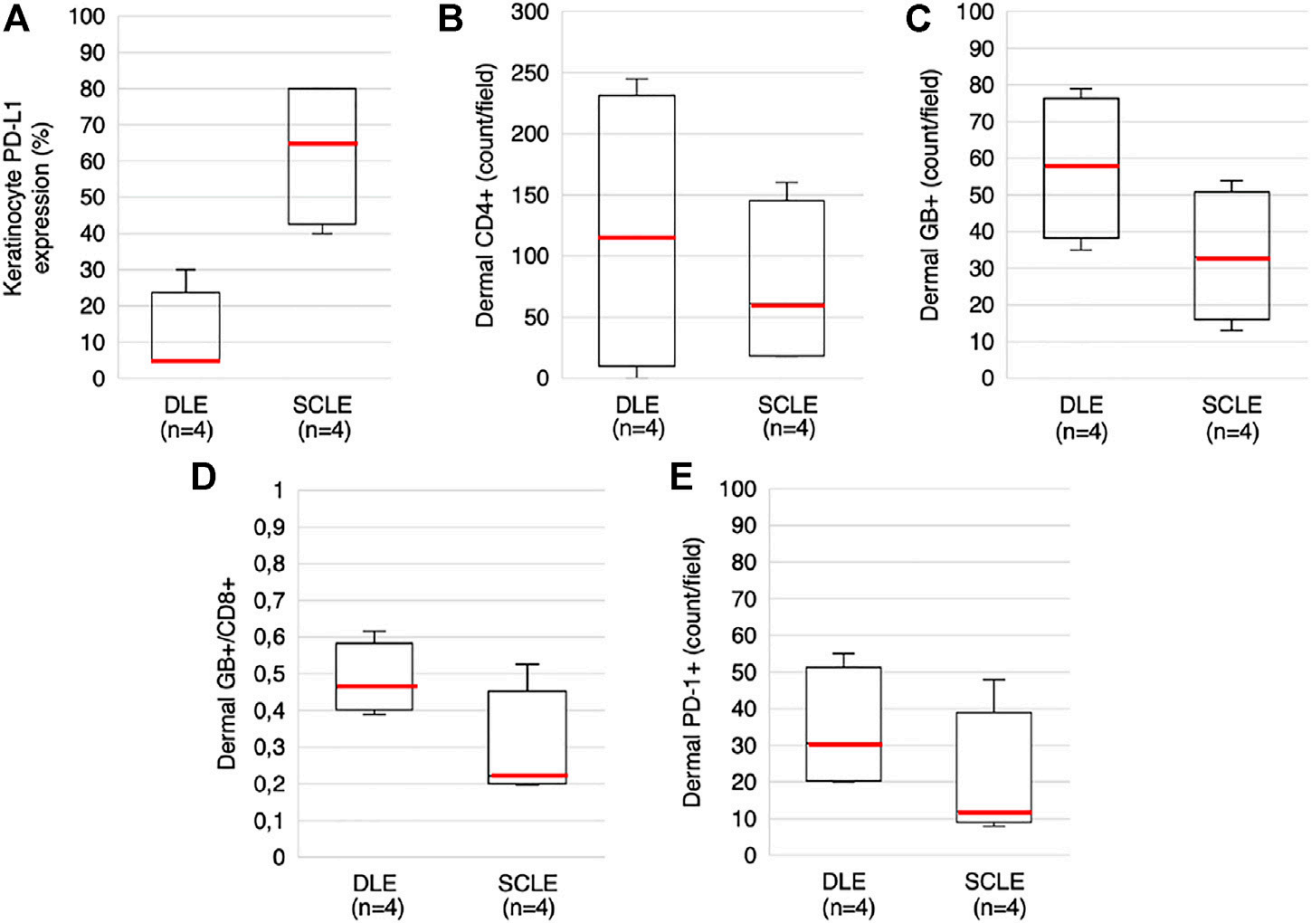
Boxplots presenting KC PD-L1 expression, dermal CD4^+^, GB+, GB+/CD8^+^ and PD-1+ cell counts in DLE and SCLE groups **(A)** KC PD-L1 expression **(B)** Dermal CD4^+^ cell count **(C)** Dermal GB+ cell count **(D)** Dermal GB+/CD8^+^ ratio **(E)** Dermal PD-1 cell count. Red lines representing the median values of each. The median values of these parameters differ between the DLE and the SCLE groups (SCLE, subacute cutaneous lupus erythematosus; DLE, discoid lupus erythematosus; GB, granzyme B; KC, keratinocyte; PD-1, programmed cell death 1; PD-L1, programmed cell death ligand 1).

To analyze the differences of KC-PD-L1 expression between the DLE and SCLE groups, the percentage of the positive KC were evaluated in the samples, the medians were calculated and compared. The KC PD-L1 expression was higher in the SCLE group compared to the DLE group (65% vs. 5%, respectively) (Figure 3A). The 2 I-SCLE samples showed higher KC PD-L1 expression, than the 2 DI-SCLE samples (medians 80% vs. 45%, respectively).

The localization and composition of the dermal inflammatory cells were analyzed, the data is summarized in Table 2. CD3^+^ T lymphocytes were detected in the superficial dermis in all cases, while follicular localization was specific for DLEs. In most of the samples the CD4^+^/CD8^+^ ratio resulted <1, the dominance of CD4^+^ lymphocytes over CD8^+^ was found only in 2 DLE cases and in 1 DI-SCLE case. Counting the dermal cells, higher median values were found in the DLE group compared to the SCLE group regarding the following markers: CD4 (115 vs. 61), GB (58 vs. 33), PD-1 (30.5 vs. 12) and GB+/CD8^+^ ratio (0.46 vs. 0.22) (Figures 3B–E). No clear differences regarding the medians of other stained markers (CD3, CD8, CD123, CD163) or ratio (PD-1+/CD3^+^) were observed between the two groups. We detected CD123 positive pDCs in the dermis in DLE and SCLE cases. Furthermore, these cells formed clusters in DLE cases in comparison to SCLE samples, where this pattern was absent. The presence of dermal CD163^+^ histiocytes differed widely from 1+ to 3+.

Data regarding the dermal immunostainings of the I-SCLE and DI-SCLE samples are shown in Table 2. The number of GB+ cells in the dermis is higher in all I-SCLE cases than in the DI-SCLE cases. Cell counts differed widely among other markers.

**FIGURE 4:**
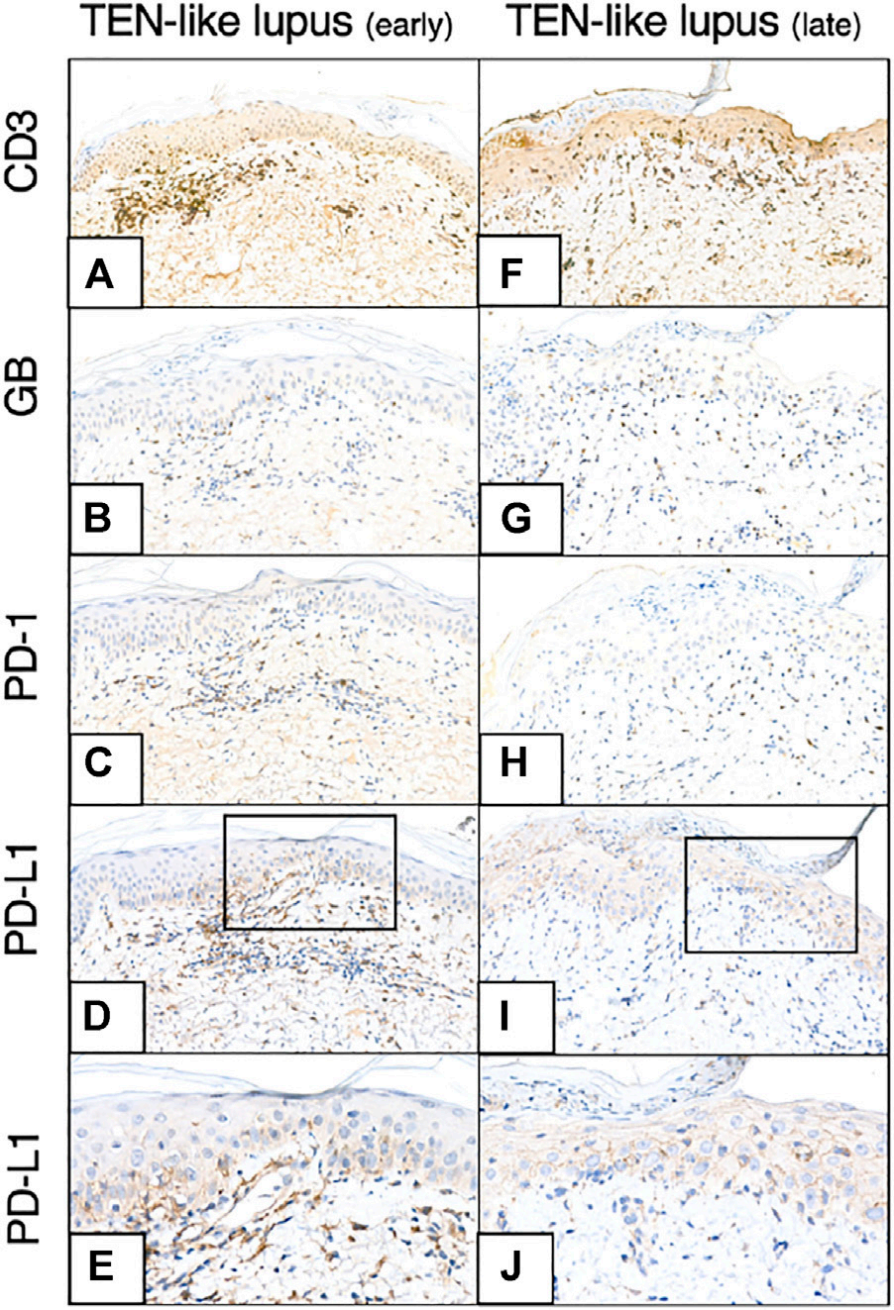
CD3, GB, PD-1, PD-L1 immunohistochemistry of earlier and later TEN-like lupus samples **(A–-E)** TEN-like lupus, early sample **(F–J)** TEN-like lupus, late sample [Magnifications: ×20, except: **(E,J)**: ×40] As the clinical picture progressed activated T-cell numbers (GB+ T-cell numbers) and KC PD-L1 expression increased (TEN, toxic epidermal necrolysis; GB, granzyme B; KC, keratinocyte; PD-1, programmed cell death 1; PD-L1, programmed cell death ligand 1).

KC PD-L1 expression and the number of GB+ cytotoxic T-cells and the GB+/CD8^+^ ratio increased (from 22 to 43 and 0,24 to 0,49, respectively) by time in the consecutive samples of the TEN-like lupus patient (Figure 4).

## Discussion

Earlier case reports of PD-1 inhibitor therapy induced autoimmune diseases suggested that these pharmaceuticals might break the peripheral tolerance or trigger a subclinical autoimmune condition into a fully developed clinical picture (5, 14–16). Here we present an immunohistochemical study comparing PD-1 inhibitor-induced SCLE, idiopathic SCLE and other CLE subtypes: DLE and TEN-like lupus erythematosus. According to the systematic review of Bolton et al. 13 cases of immune checkpoint inhibitor-induced SCLE cases can be found in the literature, 12 of these are due to nivolumab or pembrolizumab (17). To the best of our knowledge there is no data on PD-1/PD-L1 inhibitor-induced DLE. No study has explored the role of the PD-1/PD-L1 pathway neither in SCLE, nor in other subtypes.

SCLE and DLE are distinct on dermatologic examination; however, they show strong similarities regarding the immunopathogenesis and the related histological features, the presence of interface dermatitis with apoptosis and hydropic degeneration of the KCs. According to our observations regarding the KC PD-L1 expression a clear difference was found between these two entities: in the SCLE group a higher KC PD-L1 expression was observed compared to the DLE group, while healthy skin was negative. SCLE and DLE also differ regarding the dermal inflammatory infiltrate. In general, the grade of cutaneous T-cell infiltration is stronger, deeper, and more perifollicular in DLE with increased number of GB+ activated cytotoxic T-cells than in SCLE. In our clinicopathological study the dermal PD-1 staining was more prominent in the DLE group compared to the SCLE samples. This is in line with a recent investigation showing elevated expression of a newly discovered PD-1 homologous transmembrane protein (PD-1H) on T-cells in DLE skin samples (18). Immunophenotypic patterns of infiltrating T-cells (CD3, CD4, CD8, GB) and pDCs (CD123) showed close resemblance to previous observations (19, 20).

Comparing the I-SCLE and DI-SCLE patients the KC PD-L1 expression was higher in the former group. Schaberg et al. compared 5 cases of PD-1/PD-L1-inhibitor associated lichenoid dermatoses with 3 cases of non-drug related lichen planus. They found slightly elevated level of KC PD-L1 regardless of the disease origin (21). The investigated markers of the dermal inflammatory infiltrate and the PD-1/PD-L1 axis showed many similarities in the DI-SCLE cases and the I-SCLE cases, however the dermal GB+, activated cytotoxic T-cell number seems to be higher in I-SCLE.

The two samples taken from our TEN-like lupus patient represented two moments of the disease progression’s timeline. As the clinical picture of the erythematous plaques developed, an elevated KC PD-L1 expression was observed, which further increased with the exacerbation of the skin symptoms, similarly to the findings of Vivar et al. They found the same phenomenon in two biopsy samples collected at different time points in a drug-induced TEN patient (22). The possible regulatory role of PD-1/PD-L1 axis in the interaction between KCs and lymphocytes has been studied before (23–25). Prior research using cultured epithelial KCs isolated from the oral cavity of healthy patients showed increasing PD-L1 expression after stimulation with proinflammatory cytokines (mainly interferon-γ (IFNγ)). On the other hand, inhibiting PD-L1 expression by specific antibody resulted proliferative responses of the co-cultured allogenic T-cells and increased IFNγ production (24). Other authors described the regulatory functions of PD-L1 expressing murine KCs on activated T-cells. They concluded that KC-associated PD-L1 expression can directly downregulate the CD8^+^ cytotoxic T-cells and decrease the cytotoxicity as a defense mechanism (23, 25). These findings suggest that alterations in the regulation of the PD-1/PD-L1 axis might influence the development of skin autoimmunity and that the increasing PD-L1 expression on the KCs parallel with the progression of the clinical picture might serve as a natural defense mechanism against immune attack in a hyperacute reaction.

Only slight elevation of KC PD-L1 expression was observed in our DLE samples. This finding is congruent with previous results described in chronic inflammatory skin diseases with interface dermatitis other than DLE, where the authors found only mild to moderate elevation of KC PD-L1 positivity. Costa et al. recently found low KC PD-L1 expression in their study investigating oral lichen (26).

In summary, our is the first study exploring the expression of the immune checkpoint elements (PD-1/PD-L1) in CLE. Our results showed higher KC PD-L1 staining in the subacute form of CLE compared to the low KC PD-L1 expression seen in DLE, a chronic subtype of CLE. Clinicopathological and immunohistochemical similarities between PD-1 inhibitor-induced SCLE and idiopathic SCLE were demonstrated, however slight differences were also found. A more detailed way to further investigate this scientific area would be in our interest in the future, although the rarity of PD-1 inhibitor induced SCLE is a main limiting factor. Alterations of the PD-1/PD-L1 axis seems to play a role in the pathogenesis of CLE.

## Data Availability

The original contributions presented in the study are included in the article/supplementary material, further inquiries can be directed to the corresponding author.
